# Evaluation of a voucher scheme to increase child physical activity in participants of a school physical activity trial in the Hunter region of Australia

**DOI:** 10.1186/s12889-021-10588-0

**Published:** 2021-03-23

**Authors:** Kathryn Reilly, Adrian Bauman, Lindsey Reece, Christophe Lecathelinais, Rachel Sutherland, Luke Wolfenden

**Affiliations:** 1 School of Medicine and Public Health, Faculty of Health and Medicine, University of Newcastle, Callaghan, NSW 2308 Australia; 2 Hunter New England Population Health, Wallsend, NSW 2287 Australia; 3 Hunter Medical Research Institute, New Lambton, NSW 2305 Australia; 4 SPRINTER research group, Prevention Research Collaboration, Sydney School of Public Health, Faculty of Medicine and Health, The University of Sydney, Camperdown, NSW 2006 Australia

**Keywords:** Physical activity, Children, Organised sport, Financial incentive, Public policy

## Abstract

**Background:**

Global population data suggest that physical activity levels in children remain unacceptably low. Improved participation in organised sport has been recommended by the WHO as one strategy to improve population levels of physical activity. In 2018, in the state of New South Wales, Australia, the government introduced the Active Kids scheme, to help families meet the cost of getting children into organized sport. The aim of this study is to describe the uptake of Active Kids and assess the impact of the scheme on organized sport participation and child physical activity in a region of New South Wales.

**Methods:**

A pragmatic longitudinal study was undertaken of parents/carers from primary school aged children (5–12 years) in the Hunter region of NSW, Australia. Baseline data were collected between Oct-Dec 2017, with follow-up 12 months later. A telephone survey occurred at both time points, asking questions regarding registration and redemption of an Active Kids voucher for their child, child participation in organised sports and child physical activity levels.

**Results:**

Of the 974 parents/carers who consented to participate, 511 (52.5%) completed the telephone surveys at both time points. A very large proportion of children (*n* = 454, 89.0%) were reported by their parents/carers at baseline as meeting the minimum physical activity guideline of 60 min per day. Of participating parents/carers in this study, 407 (96.0%) reported redeeming an Active Kids voucher. Children who redeemed a voucher had three times the odds to participate in organized team sports from baseline to follow-up (*p* = 0.009). Sub group analyses identified that females who redeemed a voucher had four times the odds to participate in organized team sports (*p* = 0.012).

**Conclusions:**

Given the already active nature of this sample, no significant improvements in physical activity levels were noted, but the positive contribution community sport can have on health and wellbeing amongst children is reinforced. Whilst voucher schemes can address financial burdens across all socio-economic groups, more population targeting is needed to deliver voucher schemes to the most disadvantaged and inactive segments of the population in order to increase physical activity.

**Supplementary Information:**

The online version contains supplementary material available at 10.1186/s12889-021-10588-0.

## Background

The health benefits of physical activity during childhood and adolescence are well documented and include improvements in blood pressure, metabolic syndrome, adiposity, skeletal and mental health and psycho-social wellbeing [1]. Despite this, global population data suggest that physical activity levels in childhood remain unacceptably low, with between 15 and 38% of children aged 5–12 years meeting physical activity guideline recommendations in countries such as Australia [2], the United States (U.S.) [3] and the United Kingdom (U.K.) [4]. Additionally, it has been reported that 81% of adolescents (11–17 years) globally do not meet the World Health Organizations (WHO) global recommendations on physical activity [5].

Improved participation in organised sport has been recommended by the WHO ‘Global Action Plan on Physical Activity 2018–2030’ [6] as one strategy to improve population levels of physical activity, and one that may also accrue a range of other positive psychosocial outcomes for children [7]. Analysis by the Active Healthy Kids Global Alliance of data from 38 countries across six continents, for example, reported that just 40–59% of children participate in organised sport, providing considerable scope for improvement [8]. Research suggests that while there are a number of barriers to youth sports participation, cost of participation is frequently cited [9–13].

Government policies and programs to reduce the financial burden of organized sports participation has been recommended to improve community engagement in physical activity [14]. Accordingly, governments have introduced policies and programs supporting the introduction of financial incentives and/or vouchers for organized sports participants. Rigorous assessment of the effects of such government programs, however, represents a considerable challenge [15, 16]. Programs may be introduced before baseline measures to assess impact can be undertaken and the use of control groups is often unfeasible for the population wide schemes. Evaluations of public health policies and programs are often not undertaken, and when they are, may be opportunistic, and seek to undertake the most rigorous evaluation possible given the constraints of the practice based evaluation [15, 16].

As such, reviews have identified only a handful of evaluations of financial schemes to improve physical activity [17, 18]. A review by Molema and colleagues (2016) investigating the effectiveness of financial incentives in the healthcare setting to improve physical activity levels, found only three studies, one of which provided a free sports facility membership, one provided vouchers for one leisure centre activity and the third involved a six week exercise schedule [18]. The authors suggest the identified studies are largely of poor quality/design and the outcomes suggest that the impact is small and short term [18]. Another financial incentive example is the Canadian Child Fitness Tax Credit (CFTC) [19] which was introduced to assist families with the costs of registering a child under the age of 16 years in organized physical activity programs, providing a tax credit of $75 per eligible child [19]. Evaluation of the initiative of 1004 parents with children aged 2–18 years, found just over half (55.5%) of parents were aware of the CFTC and only a quarter (26.1%) claimed the CFTC in 2007 [19]. Furthermore, approximately half of parents (54.4%) surveyed stated their child was enrolled in organized physical activity and of those who claimed the CFTC in 2007, only 15.6% agreed the CFTC had increased their child’s participation in organized physical activity [19]. The results of the study also indicated that wealthier families appeared to have benefited more from the tax credit scheme than lower income families [19].

In Australia, several states and territories have introduced voucher schemes to improve physical activity levels for various target populations including youth, women and girls and disadvantaged and remote communities [20]. Whilst uptake of such voucher schemes appears to be substantial, rigorous evaluation of impact on child physical activity is lacking [20]. A recent study (2020) [20] found Australian parents report a median annual expenditure for child physical activities of $447 (IQR $194.2–936). The median voucher value across the five out of eight Australian State and Territories who have implemented a voucher scheme was found to be AU$150 per annum (range AU$50–200 per annum) [20]. In New South Wales (NSW), a state with a geographical and socio-economical diverse population (40% of the state’s population live in regional areas) of approximately eight million people, the state government introduced the Active Kids (AK) scheme in 2018. The AK scheme is a four year investment of greater than $200 million, to help families across the entire state meet the cost of getting children into organized sport and recreation activities [21]. The AK scheme aims to reduce the financial barrier to child organized sport participation and help increase the physical activity behaviours of children 4.5 to 18 years of age. In 2018, parents/carers of school-enrolled children in NSW were able to register for one $100 AK voucher valid for redemption throughout the calendar year, which was only to be claimed through an online government platform. Vouchers can only be redeemed through an accredited provider authorised by NSW government. Physical activity providers eligible to register as an ‘accredited AK provider’ are those that provide activities including participation in moderate intensity organised sport, lasting a minimum of eight weeks. This includes sporting clubs and associations affiliated with a recognised State Sporting Organisation located in NSW, and for-profit and not-for-profit activity providers in NSW.

The introduction of AK in NSW represents the largest investment by a state government in Australia to date to improve child organized sport participation [21]. Given considerable investments by governments, there is a need for pragmatic, population-level program evaluations to assess the impact of population organized sport and physical activity population programs, like that which is underway in NSW [21]. During the first year of the AK scheme we undertook an opportunistic study to assess the potential impact of the NSW AK scheme in the Hunter region of NSW in a large group of schoolchildren who had previously participated in a child school-based physical activity randomised control trial [22]. Specifically, the research team sought to; i) describe the reach, uptake and use of AK by child demographic characteristics, previous organized sport participation and physical activity levels; ii) describe parental/carer attitudes towards the AK scheme; iii) and assess the impact of the AK scheme on organized sport participation and child physical activity in the Hunter region of NSW, Australia.

## Methods

### Design and setting

To address the study objectives, a pragmatic longitudinal study was undertaken of parents/carers from primary school aged children (5–12 years) in the Hunter region of NSW, Australia. Baseline data used in this study were collected following their engagement in a previous trial between Oct-Dec 2017. Follow-up data collection took place in Oct-Dec 2018. Approval to conduct the study was obtained from Hunter New England Area Health Service Human Research Ethics Committee (no. 06/07/26/4.04), the University of Newcastle (H-2008-0343), the University of Newcastle (Ref. No. H-2008-0343) and the Maitland-Newcastle Catholic Schools Office.

### Sample and recruitment

The study used a non-probability sampling procedure whereby we opportunistically draw on a convenience sample of parents/carers of primary school aged children (5–12 years of age) who had previously agreed to be part of a research ‘panel’ and approached to participate in future research studies. The panel was established at the end of a randomised trial of a school-based healthy eating and physical activity intervention which included 12 randomly selected Catholic Schools in the Hunter Region of NSW, Australia [22]. The panel was established to provide a pool of potential participants for future studies/surveys conducted by the research team. Ninety-nine percent of parents/carers participating in the randomised trial agreed, via a telephone survey, to be a member of the research panel. All panel members were sent information statements and consent forms, telephoned by the research team and invited to participate in this study. There were no additional eligibility criteria to participate in the current study.

### Data collection and measures

Trained interviewers conducted a computer-assisted telephone survey with participating parents/carers at two time points; i) Baseline: Pre-AK scheme (Oct-Dec 2017); and ii) Follow-up: approximately 10–12 months following the AK launch (Oct-Dec 2018). Computer assisted telephone interviews ensured standardised administration of the survey. The survey was developed by the research team and piloted prior to study use for comprehension in a sample of parents of primary school aged children. Survey items were not under license and a full list is provided as Supplementary Material 1.

#### Child/parent/carer characteristics

Child demographic data including gender, school year and postcode of residence were previously collected from panel participants. If parents/carers had multiple students within the target age range, a target student was randomly selected. Additionally, parent/carer education level and current employment status was collected during the telephone survey conducted with parents/carers at baseline (Oct-Dec 2017) using items from the NSW Population Health Survey Questionnaire 2017 [23].

All participating parents/carers were asked; “In the last 12 months, how much did you pay in total for the targeted child on structured physical activity and sport? This includes all activities including the activity where you used an AK voucher and all other structured physical activity and sport” (possible responses were; 1. <$50, 2. $50–$149, 3. $150–$299, 4. $300+, 5. Don’t know).

#### Registration and redemption of AK voucher

During the follow-up telephone survey (Oct-Dec 2018) parents/carers were asked if they had; i) registered the target child for AK and; ii) if they had redeemed the AK voucher for the target child to participate in an organized team or individual sport. Registering for a voucher required parents to create an account on the NSW government ‘My Service NSW’ website [24] and enter child details on the AK application page. A voucher number was then generated for each individual child application. Redeeming a voucher required parents/carers to locate an eligible AK sport provider and provide the voucher number at registration/payment time through the providers preferred process. The voucher entitled redeemers to a $100 discount off organized sport fees. For parents/carers who had redeemed a voucher, they were asked to report the month in which that occurred. Parents/carers were also asked how many (if they were the parent of more than 1 child of primary school age) children they had registered a voucher for.

#### Parent/carer attitudes regarding the AK scheme

Parents/carers who reported redeeming an AK voucher for their target child were asked during the telephone survey at time point 2 (Oct-Dec 2018) to report their level of agreement to a series of statements using a 4-point Likert scale. Responses ranged from ‘strongly agree’ to ‘strongly disagree’. Specifically parents/carers were asked their level of agreement with the following statements: a) I would not have enrolled my child in organized sport without the support of the AK scheme; b) Finding an activity or sporting group that accepted the AK voucher was difficult; c) The AK voucher supported my child to try new organized sports; d) It was easy to redeem the AK voucher; and e) I support the continuation of the AK scheme.

Additionally, parents/carers who reported redeeming an AK voucher for their child were asked the main reason they had registered with the AK scheme (possible responses were; 1. Money/financial support for activity, 2. Support an activity the child usually participated in, 3. Try a new activity, instead of usual chosen sport, 4. Try a new activity in addition to usual chosen sport, 5. Join an activity the child’s friends participate in, 6. To encourage the child to be more physically active, 7. Improve child’s fitness, 8. Manage child’s weight (overweight/obesity), 9. Improve performance in activity, 10. Other/don’t know).

#### Participation in organized team or individual sports

Organized sport participation was measured using two items from the Longitudinal Study of Australian Children (LSAC) parent-report questionnaire [25] pertaining to the targeted child’s regular participation in organized team and individual sports. During the telephone survey at both time points, parents/carers were asked if in the last 12 months their child had regularly participated in; i) organised team sports and/or; ii) organised individual sports. Those that responded ‘yes’ to either of these items were also asked what sport their child participated in. Examples of team sports include soccer, rugby league, rugby union, netball and basketball. Examples of individual sports are swimming, gymnastics, golf, martial arts and tennis.

#### Physical activity outside of school hours and on weekends

As AK targets participation in organized sport that occurs outside of school hours, parents/carers were asked how many hours their child was physically active; i) outside school hours on weekdays, and; ii) on weekends, using a four item measure from the NSW Population Health Survey Questionnaire 2017 [23].

Additionally, parents/carers who reported redeeming an AK voucher for their child were asked; “In your opinion how has AK influenced your child’s total time being physically active?” (Possible responses were; 1. Increased the child’s activity a lot; 2. Increased the child’s activity slightly; 3. The child’s activity stayed about the same; 4. Decreased the child’s activity slightly; 5. Decreased the child’s activity a lot; 6. Not sure).

### Analysis

All analyses were performed in SAS 9.3 (SAS Institute Inc., Cary, NC) in 2019. Descriptive statistics were used to describe student and parent level characteristics. Parent/carer postcodes were used to classify children as residing in ‘higher socio-economic areas’, if their post-code was ranked above the median of NSW postcodes based on the Socio-Economic Indexes For Australia (SEIFA) database [26]. Post codes were also used to categorize child residential locality as either ‘rural’ (those that resided in outer regional, remote and very remote areas) or ‘urban’ (those that resided in inner regional or major cities) based upon the 2016 Accessibility/Remoteness Index of Australia (ARIA) [27].

Parent perceptions of the AK intervention were measured both dichotomously and by grouping. For questions relating to parent perceptions of the AK scheme and child experience with organized sport, responses were dichotomised into ‘Agree/Strongly agree’ and ‘Disagree/Strongly disagree/don’t know’. For the question regarding parent’s/carer’s perception of child level of physical activity being influenced by AK, responses were grouped into: 1. Increased child’s physical activity a lot; 2. Increased child’s physical activity slightly; 3. Child’s physical activity stayed the same; and 4. Decreased child’s physical activity / don’t know.

To assess whether there was a significant change over time, Mixed Effects Logistic Regression Models were used on the two dichotomous outcomes: participation in organized team (yes/no), and participation in organized individual sports (yes/no). Total number of hours of physical activity outside of school was derived by combining the separate responses for number of hours of physical activity outside of school during the weekday and weekend. To assess whether those continuous physical activity outcomes changed over time from baseline (pre-AK scheme), to follow-up (post-AK scheme launch). Mixed Effects Linear Regression Models were used. All models included a random intercept effect for school, as well as a random nested intercept for child, to account for potential clustering effects. All models included a fixed effect for time and controlled for child gender, child school year and socio-economic status (SES) where appropriate.

Additionally, similar Mixed Effects Linear Regression Models were used to assess whether there was a differential effect on continuous physical activity outcomes between AK voucher redeemers and non-redeemers over time by including a time by redeeming (yes/no) interaction fixed effect.

#### Subgroup analysis on voucher redemption

As child gender, low physical activity level and low SES have previously been found to influence child participation in physical activity [28, 29], subgroup analyses were performed incorporating these characteristics by including a three-way interaction term (time by redeeming by subgroup) in each of the models. Low physically active children were defined as those whose parents/carers reported at baseline that their child was not meeting the national guideline of a minimum of 60 min of physical activity per day [30]. High physically active children were those whose parents/carers reported they did meet the minimum 60 min of physical activity per day. SEIFA rankings based on household postcode were used to define children as low (bottom 20% of participants) and high (remaining 80% of participants) SES.

## Results

### Child/parent/carer characteristics (Table 1)

Of the 974 parents/carers who consented to be panel members, 804 (82.5%) completed the pre-AK survey. Of these 224 (23.0%) could not be contacted, 53 (5.4%) refused and 511 (52.5%) completed the telephone surveys at both time points. As seen in Table 1, the majority of parents/carers were employed (*n* = 339, 68.4%) and approximately half had a university degree or advanced education (*n* = 265, 53.6%). As reported by parents/carers, 51.2% (*n* = 260) of children in the study were male, and there was an even spread of children in all class years (kindergarten to year 6; 5–12 years of age). As defined by SEIFA, a little over half of participants were categorised as residing in ‘low’ SES areas (*n* = 269, 52.8%), and no participants resided in rural locations, with the majority residing in major cities as classified by the ARIA (*n* = 418, 82.0%). A large proportion of children (*n* = 454, 89.0%) were reported by their parents/carers at baseline as meeting the minimum physical activity guideline of 60 min per day.

**Table 1:** Child and parent characteristics

Characteristic	Participants (***n*** = 511)^**a**^	Redeemed AK^**a**^ voucher ***n*** = 407 (79.6%)	Total n = 511^**b**^ n (%)
**Child Gender**^**c**^	Male	218 (53.96)	259 (51.08)
	Female	186 (46.04)	248 (48.92)
**Child School Year**^**b**^	Kindergarten	78 (19.16)	95 (18.59)
	Year 1	62 (15.23)	76 (14.87)
	Year 2	56 (13.76)	66 (13.11)
	Year 3	64 (15.72)	76 (14.87)
	Year 4	46 (11.30)	65 (12.72)
	Year 5	50 (12.29)	63 (12.33)
	Year 6	51 (12.53)	69 (13.50)
**Socio-economic Index**	Lower	276 (67.98)	342 (67.06)
	Higher	130 (32.02)	168 (32.94)
**Rural/Urban region**^**b**^	Rural	0	0
	Urban – inner regional	70 (17.24)	91 (17.88)
	Urban – major cities	336 (82.76)	418 (82.12)
**Child physical activity level**^**b**^	Meets guideline of minimum 60 mins per day	372 (91.40)	454 (89.02)
	Does not meet guideline of minimum 60 mins per day	35 (8.60)	56 (10.98)
**Parent employment status**	Employed, self-employed	352 (86.49)	439 (86.08)
	Unemployed, home duties, student, other	55 (13.51)	71 (13.92)
**Parent education level**	TAFE Certificate or Diploma or less	188 (46.19)	246 (48.24)
	University degree, Advanced Education	219 (53.81)	264 (51.76)
**Number of kids registered for an Active Kids voucher**	1	131 (32.19)	142 (31.63)
	2	191 (46.93)	206 (45.88)
	3	73 (17.94)	78 (17.37)
	4	12 (2.95)	13 (3.79)
**When Active Kids voucher redeemed**	January – June 2018	328 (80.59)	328 (80.59)
	July – December 2018	56 (13.76)	56 (13.76)
	Don’t know	23 (5.65)	23 (5.65)
**Money spent on child physical activity and sport in the last 12 months.**	<$50	0 (0.00)	13 (2.55)
	$50–$149	10 (2.46)	17 (3.33)
	$150–$299	50 (12.29)	62 (12.16)
	$300+	344 (84.52)	412 (80.78)
	Don’t know	3 (0.74)	6 (1.18)
**Why registered with Active Kids (*n*** **= 424)**	Money/financial support for activity	335 (82.31)	351 (82.78)
	Support an activity the child usually participated in	45 (11.06)	45 (10.61)
	Try a new activity, instead of usual chosen sport	2 (0.49)	2 (0.47)
	Try a new activity in addition to usual chosen sport	4 (0.98)	4 (0.94)
	Join an activity the child’s friends participate in	2 (0.49)	2 (0.47)
	To encourage the child to be more physically active	5 (1.23)	5 (1.18)
	Improve child’s fitness	0 (0.00)	1 (0.24)
	Manage child’s weight (overweight/obesity)	2 (0.49)	2 (0.47)
	Improve performance in activity	2 (0.49)	2 (0.47)
	Other (Please specify)	9 (2.21)	9 (2.12)
	Don’t know	1 (0.25)	1 (0.24)

^a^ AK = Active Kids

^b^Missing data from 1 participant

^c^Missing data from 3 participants

There were no significant differences in SES, rurality, employment status or education level between those parents/carers who completed the two surveys and those that did not.

### Registration and redemption of AK voucher

Of the 511 participating parents in this study, 424 (96.6%) reported they had registered for an AK voucher for their child in the last 12 months of which 407 (96.0%) reported they had redeemed the voucher at the time of the follow-up telephone survey (Oct-Dec 2018). Further information regarding voucher redemption is found in Table 1.

### Parent/carer attitudes of the AK scheme (Table 2)

The majority of parents/carers found it easy to find an accredited provider that accepted the AK voucher (*n* = 378, 92.9%) and to redeem the AK voucher (*n* = 379, 93.0%). Almost all parents/carers supported the continuation of AK (*n* = 403, 99.0%). Only 11% (*n* = 45) agreed they would not have enrolled their child in a sporting activity without the support of AK. Almost a third of parents/carers agreed AK supported their child to try a new organized sport (*n* = 124, 30.5%). The majority of parents/carers (*n* = 304, 74.7%) perceived the redemption of the AK voucher did not change the amount of physical activity their child participated in (n = 304, 74.7%) and stated that money/financial support for physical activity was the main reason they registered for a voucher (*n* = 351, 82.8%). Additional information regarding parent/carer attitudes is found in Table 2.

**Table 2:** Parent perceptions and attitudes towards the Active Kids scheme

1. Parent attitudes towards the Active Kids scheme n = 407^**a**^	Agree/ Strongly agree n (%)		Disagree/ Strongly disagree/ Don’t know n (%)	
I would not have enrolled my child in organized sport without the support of the Active Kids scheme.	45 (11.0)		362 (89.0)	
Finding an activity or sporting group that accept the Active Kids voucher was difficult.	29 (7.1)		378 (92.9)	
Active Kids supported my child to try new organized sports.	124 (30.5)		283 (69.5)	
It was easy to redeem the Active Kids voucher.	379 (93.1)		28 (6.9)	
I support the continuation of the Active Kids scheme.	403 (99.0)		4 (1.0)	
**2. Parent perceptions of child organised sport participation** **n = 511**^**b**^	**Agree/ Strongly agree** **n (%)**		**Disagree/ Strongly disagree/ Don’t know** **n (%)**	
My child enjoys playing organized sport.	483 (94.7)		27 (5.3)	
My child has made new friends through participating in organized sport.	466 (91.4)		44 (8.6)	
My child is likely to play organized sport next year.	490 (96.1)		20 (3.9)	
**3. Change in child physical activity – parent perception** **n = 407**^**a**^	**Increased child activity a lot** **n (%)**	**Increased child activity slightly** **n (%)**	**Child’s activity stayed the same** **n (%)**	**Decreased child’s activity/don’t know** **n (%)**
Parent perceived change in child physical activity	42 (10.3)	57 (14.0)	304 (74.7)	4 (1.0)

^a^Parents who redeemed the Active Kids voucher

^b^Missing data for 1 participant

### Participation in organized team or individual sports (Table 3)

Children who redeemed an AK voucher had three times the odds as those that did not redeem a voucher to participate in organized team sports from baseline to follow-up (relative OR = 3.11; 1.41–6.87, *p* = 0.009). Additionally, sub group analyses identified that females who redeemed the AK voucher had four times the odds as those females that did not redeem a voucher to participate in organized team sports (relative OR = 4.40; 1.51–12.84, *p* = 0.012). There was no significant difference in participation in organized individual sports between those that did or did not redeem an AK voucher across the study period (relative OR = 1.16; 0.54–2.48, *p* = 0.68).

**Table 3:** Changes in child participation in organised team and individual sports and redemption of the Active Kids voucher – adjusted for child gender, class year and socio-economic status where applicable

Participants	Participation in organised team / individual sport	Redeemed AK^**a**^ voucher Odds Ratio^**b**^ (95% Confidence Intervals)	Did not redeem AK^**a**^ voucher Odds Ratio^**b**^ (95% Confidence Intervals)	Relative Odds Ratio (95% Confidence Intervals)	***p***-value
**All children (n = 511)**	Team	1.58 [1.03;2.42]	0.51 [0.26;0.99]	3.11 [1.41;6.87]	0.009*
	Individual	0.70 [0.48;1.02]	0.61 [0.31;1.18]	1.16 [0.54;2.48]	0.68
**Males (*n*** **= 259)**	Team	1.45 [0.73;2.86]	0.71 [0.25;2.05]	2.03 [0.58;7.16]	0.24
	Individual	0.68 [0.42;1.11]	0.63 [0.22;1.84]	1.08 [0.33;3.49]	0.89
**Females (*n*** **= 248)**	Team	1.70 [0.95;3.03]	0.39 [0.16;0.95]	4.40 [1.51;12.84]	0.012*
	Individual	0.73 [0.40;1.33]	0.59 [0.25;1.41]	1.23 [0.43;3.52]	0.68
**Low active children**^**c**^ **(*n*** **= 56)**	Team	1.77 [0.41;7.69]	0.79 [0.16;3.98]	2.24 [0.25;19.92]	0.41
	Individual	0.67 [0.20;2.28]	0.35 [0.07;1.67]	1.94 [0.26;14.17]	0.46
**High active children**^**d**^ **(n = 454)**	Team	1.56 [0.96;2.54]	0.44 [0.19;0.99]	3.56 [1.37;9.23]	0.016*
	Individual	0.70 [0.46;1.08]	0.71 [0.32;1.58]	1.00 [0.40;2.48]	0.99
**Low SES children**^**e**^ **(*n*** **= 143)**	Team	1.30 [0.54;3.12]	0.35 [0.09;1.36]	3.73 [0.74;18.87]	0.09
	Individual	0.95 [0.43;2.10]	1.00 [0.28;3.57]	0.95 [0.21;4.25]	0.93
**High SES children**^**f**^ **(*n*** **= 104)**	Team	1.00 [0.28;3.61]	1.50 [0.32;7.13]	0.67 [0.09;5.01]	0.64
	Individual	0.74 [0.28;1.92]	1.00 [0.16;6.07]	0.74 [0.10;5.68]	0.73

^a^Active Kids

^b^Odds ratio from baseline to follow-up

^c^Children not meeting physical activity guideline of minimum 60 min per day

^d^Children who meet the physical activity guideline of minimum 60 min

^e^Children in the bottom 20% of participants according to SEIFA ranking

^f^Children in the top 20% of participants according to SEIFA ranking

^*^Statistically Significant

### Physical activity outside of school hours and on weekends (Table 4)

Table 4 shows changes in physical activity levels of children who did and did not redeem an AK voucher during 2018. There was a non-significant decrease in physical activity levels between the two time points, whether a voucher was redeemed or not, for physical activity after school (weekdays) (Adjusted Mean Difference (AMD) = − 0.47; − 2.47-1.52, *p* = 0.61), physical activity on weekends (AMD = − 0.92; − 2.12-0.28, *p* = 0.12) and when both combined (AMD = − 1.42; − 4.28-1.43, *p* = 0.30). Sub group analyses showed a non-significant increase in physical activity levels outside of school hours in low active children in both the voucher redeemers and non-redeemers (AMD = 1.29; − 6.33-8.91, *p* = 0.70).

**Table 4:** Changes in child physical activity and redemption of the Active Kids voucher - adjusted for child gender, class year and socio-economic status where applicable

Participants	Child physical activity (hours)	Redeemed AK^**a**^ voucher Mean Difference^**b**^ (95% Confidence Intervals)	Did not redeem AK^**a**^ voucher Mean Difference^**b**^ (95% Confidence Intervals)	Adjusted Difference (95% Confidence Intervals)	p-value
**All children** **(n = 511)**	Outside school hours (weekdays)	−1.89 [− 2.79;-0.99]	−1.42 [− 3.20;0.36]	−0.47 [− 2.47;1.52]	0.61
	On weekends	− 1.37 [− 1.91;-0.83]	− 0.45 [− 1.52;0.62]	−0.92 [− 2.12;0.28]	0.12
	Combined	− 3.23 [− 4.51;-1.95]	−1.81 [− 4.36;0.74]	−1.42 [− 4.28;1.43]	0.30
**Males (n = 259)**	Outside school hours (weekdays)	−1.60 [− 2.84;-0.37]	− 0.65 [− 3.52;2.22]	−0.95 [− 4.08;2.17]	0.51
	On weekends	− 1.48 [− 2.22;-0.73]	−0.23 [− 1.96;1.49]	−1.24 [− 3.12;0.64]	0.17
	Combined	−3.07 [− 4.84;-1.31]	− 0.81 [− 4.94;3.31]	− 2.26 [− 6.74;2.22]	0.29
**Females (n = 248)**	Outside school hours (weekdays)	−2.23 [− 3.57;-0.89]	− 1.94 [− 4.25;0.38]	−0.29 [− 2.97;2.38]	0.81
	On weekends	− 1.25 [− 2.06;-0.44]	− 0.59 [− 1.99;0.81]	− 0.66 [− 2.27;0.96]	0.39
	Combined	−3.42 [−5.34;-1.50]	−2.48 [− 5.80;0.84]	−0.94 [− 4.77;2.89]	0.60
**Low active children**^**c**^ **(*n*** **= 56)**	Outside school hours (weekdays)	1.96 [− 1.28;5.20]	1.47 [− 2.72;5.65]	0.50 [− 4.79;5.79]	0.83
	On weekends	1.12 [−0.86;3.10]	0.45 [−2.09;3.00]	0.67 [− 2.56;3.89]	0.64
	Combined	3.24 [− 1.43;7.92]	1.95 [− 4.06;7.97]	1.29 [− 6.33;8.91]	0.70
**High active children**^**d**^ **(*n*** **= 454)**	Outside school hours (weekdays)	−2.26 [−3.26;-1.26	− 2.15 [− 4.27;-0.02]	−0.11 [− 2.46;2.24]	0.92
	On weekends	− 1.61 [− 2.21;-1.01]	− 0.69 [− 1.96;0.58]	− 0.92 [− 2.33;0.49]	0.17
	Combined	−3.86 [− 5.28;-2.44]	−2.78 [− 5.80;0.25]	−1.08 [− 4.42;2.26]	0.47
**Low SES children**^**e**^ **(n = 143)**	Outside school hours (weekdays)	− 3.00 [− 4.90;-1.10]	− 1.84 [− 5.38;1.69]	− 1.15 [− 5.17;2.86]	0.51
	On weekends	− 1.87 [− 3.01;-0.73]	0.46 [− 1.71;2.63]	−2.33 [− 4.78;0.12]	0.06
	Combined	−4.87 [−7.57;-2.17]	−1.26 [− 6.40;3.87]	− 3.61 [− 9.41;2.20]	0.18
**High SES children**^**f**^ **(*n*** **= 104)**	Outside school hours (weekdays)	− 2.39 [− 4.64;-0.14]	0.53 [−3.63;4.70]	− 2.93 [− 7.66;1.81]	0.18
	On weekends	−1.49 [− 2.85;-0.13]	− 1.31 [− 3.81;1.20]	− 0.18 [− 3.03;2.67]	0.88
	Combined	− 3.79 [− 7.02;-0.56]	−0.77 [− 6.71;5.16]	−3.02 [− 9.78;3.74]	0.32

^a^Active Kids

^b^Mean Difference from baseline to follow-up

^c^Children not meeting physical activity guideline of minimum 60 min per day

^d^Children who meet the physical activity guideline of minimum 60 min

^e^Children in the bottom 20% of participants according to SEIFA ranking

^f^Children in the top 20% of participants according to SEIFA ranking

## Discussion

This study assessed the potential impact of the NSW AK scheme in the Hunter region of NSW among a sample of children that had previously participated in a trial of a healthy eating and physical activity intervention. The study found that voucher registration and use was ubiquitous in this sample, parents/carers were highly supportive of the scheme, and that those that redeemed the voucher had three times the odds of organized team sports participation than those that did not. Almost 90% of parents/carers indicated that they would register their child in organized sport without the voucher, an important characteristic of the sample, suggesting very high physical activity levels of children in this group. Additionally, there were no significant differences in changes in child physical activity among those that redeemed and did not redeem a voucher. Nearly three quarters of parents/carers reported their child’s physical activity had stayed the same with redemption of the voucher. These figures are similar to those reported by Spence et al. (2012) regarding the Canadian CFTC where only 15.6% agreed the CFTC has increased their child’s participation in organised physical activity [19]. Unsurprisingly, the majority of vouchers were redeemed in the first half of the year, soon after the scheme was released, a period when registrations occur for both summer and winter sports.

Previous research has suggested tailoring schemes to increase child physical activity on factors such as low family income, living regionally or remotely, having Middle-Eastern Asian cultural background and female gender [12, 31]. Our data does not substantiate all these, however there is some suggestion from our data that targeting low physically active children may be beneficial. There appears to be a positive, albeit non-significant, impact of the AK scheme on these children in our sample. Whilst a voucher scheme such as the AK may address some barriers to sports participation in identified groups, further barriers such as lack of access to facilities and sporting groups, and travel/transport may need to be addressed to increase the effectiveness of a stand-alone financial incentive.

A further unique characteristic of the study sample was the physical activity levels of children. The majority of parents/carers (89%) in this study reported their child met the physical activity guideline of a minimum of 60 min per day prior to the AK launch, suggesting a ceiling effect, where irrespective of the voucher, there was little room for improvement in child measures of physical activity. The high levels of child physical activity is likely to reflect the children’s participation in the prior successful school-based physical activity program, or selection bias. The unexpectedly high rates of reported child physical activity, coupled with high AK voucher redemption reduced study variability and power to undertake statistical analyses of associations, despite a relatively large study sample. It has also increased the risk of regression to the mean, which may explain the magnitude of the absolute reduction in measures of physical activity change over time. While such limitations are common in opportunistic and pragmatic policy evaluations [15], further research in other jurisdictions which address them is warranted to verify the study findings. Whilst children in this study were already physically active, the role of organised sport and the additional social and emotional benefits these sports can bring [7], provides opportunity for the AK scheme amongst these children.

Whilst AK has achieved population reach across NSW substantially supporting the financial costs towards organized sport and physical activity participation, inequalities exist within program reach and voucher use. Such findings reflect a substantial proportion of children living in disadvantaged areas have not engaged with the program, with parents/carers in the most disadvantaged quartile twice as likely to have never heard of the AK scheme and more than twice as likely to have registered for a voucher but not redeemed a voucher compared to the least disadvantaged quartile [32]. These results compare to those from the Canadian CFTC study which found 28.2% of parents in the lowest income quartile claimed the CFTC compared to 55% in the highest income quartile [19]. The findings from the current study demonstrate the high levels of population wide awareness and uptake of the AK scheme, however, further targeted work is required to increase the awareness and engagement in AK amongst socially disadvantaged groups.

Additionally, all participants in this study were previously recruited to a study involving school based physical activity strategies to increase child physical activity whilst at school [22]. This pre-existing trial may have already induced changes to child physical activity and sport participation during school. Parents/carers appeared to participate in the scheme for economic reasons, as a cost subsidy, in this already active group of children. These differences raise the issue of external validity across the state (NSW) as a whole and highlights the challenges in undertaking opportunistic regional evaluations of large-scale initiatives to improve population levels of physical activity. On a positive note, parents/carers reported it was easy to access a sporting group and redeem the voucher and perhaps unsurprisingly, supported the continuation of the voucher. Almost no parents/carers however reported registering for a voucher to encourage their child to be more physically active (*n* = 5, 1.2%).

The study findings need to be considered and interpreted in the context of methodological shortcomings. Potentially most profound was the opportunistic use of a research panel. The boutique nature of the research panel limits the external validity of the study findings and may explain, in part, a number of unexpected findings. The study sample comprised participants who had previously participated in a physical activity intervention [22], and whose parents/carers were far more educated (52% with a University degree/Advanced education) than the study region (Hunter region) (11%), and NSW more broadly (23%) [33]. Children in this sample were also reportedly more physically active with 89.0% meeting guidelines compared to 31.7% of those in the study region and 24.2% in NSW [34]. More than half (54%) of the eligible NSW population registered for an AK voucher in 2018, with a state redemption rate of 81.9% [35]. Once again the redemption rate in this sample is higher than the state at 96.0% (407 out of 424 participants who registered for an AK voucher).

There are a number of limitations alongside lack of generalisability that are worth noting. This sample included only primary school age children, as research suggests that adolescent’s physical activity participation is likely to be lower, the impact of AK may also be different in this age group. Child physical activity levels were parental-reported, not objectively measured. Further, we did not split analyses of physical activity levels into in-school and out-of-school activity, limiting the identification of when children are choosing to be active. Therefore, to gain a better understanding of the impact of AK on child and adolescent physical activity levels, further research across all age groups, with the inclusion of objectively measured physical activity, such as the use of accelerometers, separating in-school and out-of-school activity is recommended. Strengths of the study include its relatively low rate of attrition and the use of a longitudinal study design, including pre-AK scheme data collection, capturing comprehensive information to assess a range of aspects of the AK scheme.

## Conclusion

This study aimed to understand the sport and physical activity behaviours of children in the Hunter region, including their awareness and uptake of the NSW government AK scheme. Whilst most of the sample of children within this study already achieved recommended levels of physical activity, those who redeemed a voucher were slightly more likely to participate in organised individual and team based activities. AK also provides a unique platform to learn more about the participation behaviours of children and adolescents and through the consistent standard metrics of the program evaluation, comparisons can be made across the state. Given the already active nature of this sample, no significant improvements in physical activity levels were noted, but the positive contribution community sport can have on the health and wellbeing outcomes amongst children and young people is reinforced. Whilst voucher schemes can help address financial barriers, and are highly supported by parents/carers, future direction should address targeting these vouchers to populations that are culturally diverse, inactive, and disadvantaged to better promote health and physical wellbeing.

## Supplementary Information

**Additional file 1.**

## Data Availability

The datasets used and/or analysed during the current study are available from the corresponding author on reasonable request.

## Abbreviations

U.S: United States
U.K: United Kingdom
WHO: World Health Organisation
CFTC: Canadian Child Fitness Tax Credit
NSW: New South Wales
AK: Active Kids
SEIFA: Socio-Economic Indexes For Australia
ARIA: Accessibility/Remoteness Index of Australia
SES: Socio-economic status
OR: Odds Ratio
AMD: Adjusted Mean Difference
