# Supplementary material for: Evaluation of a voucher scheme to increase child physical activity in participants of a school physical activity trial in the Hunter region of Australia
Source: BMC Public Health. 2021 Mar 23;21:570. doi: 10.1186/s12889-021-10588-0 (PMC7986265; doi:10.1186/s12889-021-10588-0)
Supplement: Supplementary file 1 — Additional file 1. [file 12889_2021_10588_MOESM1_ESM.docx]

***GOOD FOR KIDS, GOOD FOR LIFE.***

**Parent Telephone Survey 2018 (P1)**

Version 5, 05/10/2018

**NOTE FOR Human Research Ethics Committee:**

- **The CATI will be scripted to incorporate and finalise appropriate skips prior to being administered**

| **Parent/guardian’s name:** |  |
| --- | --- |
| **Child’s name:** |  |
| **Date of interview:** |  |

Intro1. Hello, my name is ^_INTVR_^ and I am calling from Hunter New England Local Health District (HNELHD). I am calling to talk to [PARENT’S NAME]. Is [PARENT’S NAME] available?

*[INTERVIEWER NOTE: Don't read out response options]*

1. Speaking to that person (continue)

2. Person called to phone (continue)

3. Person not available (record on log sheet) (record call back time)

4. Time not suitable (record on log sheet) (record call back time)

5. Other (record on log sheet)

.R. Refused (record on log sheet and thank them for their time)

If Intro1=2

Intro2. Hello, my name is ^_INTVR_^ and I am calling from Hunter New England Local Health District (HNELHD). [Go to intro 3]

Intro3. I’m calling regarding the Good for Kids, Good for Life program which is running in your child’s / children’s school. At the end of last year you provided a contact number and consented to being contacted by the research team to complete a short survey. The survey includes questions about your child’s eating habits and physical activity. [Go to intro 4]

Intro4. The survey will take approximately 20 minutes. Is now a convenient time or can I ring you back?

1. Yes/Appropriate

2. No/Call back later

3. No/Declines to participate (Thank and Exit)

.R. Refused

If Intro 4=1

Intro5. Great, thank you for agreeing to take part. Any information you report in this survey will be held as strictly confidential. Individual parents/children will not be identified in any way in the reporting of results. There are no right or wrong answers. Please answer the questions to the best of your ability**.** At any time, you are able to decline providing a response to any question that you do not wish to answer. Please let the interviewer know if you do not wish to answer and they will move on to the next question.

Begin. Do you have any questions before we begin the survey?

*[INTERVIEWER NOTE: - Answer as best as you can, based on the training, please do not provide information outside of the info covered in training]*

These questions will be asked about [randomly selected child] participating in the study

PHYSICAL ACTIVITY OUTSIDE OF SCHOOL HOURS

These questions are about physical activity outside of school hours. Physical activity for children includes play, games, sports, transportation such as walking or cycling to places, chores, recreation, physical education, or planned exercise.

PAOS1. How many days during the school week does [randomly selected child’s name] usually do physical activity outside of school hours? (Includes before and after school sports)

1. 5 days/week

2. 4 days/week

3. 3 days/week

4. 2 days/week

5. 1 day/week

6. Less than 1 day per week

7. Does not do any physical activity during school week [Go to PAOS6]

8. Unable to do any physical activity outside of school hrs due to health reasons [Go to PAOS8]

9. Don't know [DO NOT READ OUT]

10. Refused [DO NOT READ OUT] GO TO POAS3

PAOS2. On those days, how many hours does [randomly selected child’s name] usually do physical activity? __________________hours

[Enter 888 if don't know; 999 if refused]

PAOS3. On how many weekend days does [randomly selected child’s name] usually do physical activity?

1. 2 days/week

2. 1 day/week

3. Less than 1 day per week

4. Does not do any physical activity during weekends [Go to PAOS5]

5. Unable to do any physical activity outside of school hrs due to health reasons [Go to PAOS5]

6. Don't know [DO NOT READ OUT] [Go to PAOS8PAOS5]

7. Refused [DO NOT READ OUT] [GO TO PAOS5]

PAOS4. On those days, how many hours does [randomly selected child’s name] usually do physical activity? __________________hours

[Enter 888 of don't know; 999 if refused]

PAOS5. During 2018, has [randomly selected child] regularly participated in organised team sport (e.g. football, cricket or netball)? By ‘regularly’, we mean participating at least once per week for three months or more.

1. Yes

2. No GO TO PAOS7

3. Unable to take part in any sport due to medical/health reasons/disability GO TO PAOS7

4. Don't know [DO NOT READ OUT] GO TO PAOS7

5. Refused [DO NOT READ OUT] GO TO PAOS7

PAOS6. If Yes, which team sports has [randomly selected child] regularly participated in 2018?

***Interviewer note: Read all – choose as many as required***

1. Aerobics

2. Australian Rules Football

3. Oz tag

4. Basketball

5. Baseball/softball

6. Cheerleading

7. Cricket

8. Dancing/Ballet

9. Futsal

10. Hockey

11. Indoor sports (cricket, netball, soccer)

12. League tag

13. Martial arts

14. Netball

15. Physie (Physical Culture)

16. Rugby League

17. Rugby Union

18. Soccer

19. Touch Football

20. Water polo

21. Other (specify)

22. Did not play any sport

23. Don't know [DO NOT READ OUT]

24. Refused [DO NOT READ OUT]

PAOS7. During 2018, has [randomly selected child] regularly participated in organized individual sport (e.g. tennis, karate or gymnastics)?1. Yes

2. No [GO TO PAOS9]

3. Unable to take part in any sport due to medical/health reasons/disability [GO TO PAOS9]

4. Don't know [DO NOT READ OUT] [GO TO PAOS9]

5. Refused [DO NOT READ OUT] [GO TO PAOS9]

PAOS8. If Yes, which individual sports has [randomly selected child] regularly participated in 2018?

***Interviewer note: Read all – choose as many as required***

1. Aerobics

2. Athletics, track and field

3. Cross country / running

4. Crossfit

5. Cycling/mountain biking/bike riding

6. Dancing/Ballet

7. Diving

8. Golf

9. Gymnastics

10. Horse riding/Equestrian/Polo

11. Martial arts

12. Ninja Parc

13. Parcor

14. Physie (Physical Culture)

15. Skateboarding

16. Swimming

17. Rock climbing

18. Surf life saving

19. Surfing

20. Tennis

21. Yoga

22. Other (specify)

23. Did not play any sport

24. Don't know [DO NOT READ OUT]

25. Refused [DO NOT READ OUT]

PAOS9. What is the biggest barrier to [randomly selected child] participation in organized sport?

***Interviewer note: Read all – choose as many as required***

1. Time commitment required (work commitments, other children’s after school activities)

2. Financial cost of participation (uniforms, equipment, registration fees)

3. Lack of time to transport

4. Lack of transport

5. Fear of injury (sport is too rough)

6. Unwelcoming sporting environments at clubs

7. Lack of choice/variety of available sports / desired sport not available in my local area

8. Lack of support from friends (friends do not play sport)

9. Child does not enjoy playing sport

10. Interferes with school work (time should be spent on study)

11. Other (please specify)

12. No known barriers

13. Don't know [DO NOT READ OUT]

14. Refused DO NOT READ OUT]

ALL GO TO PAOS10

ACTIVE KIDS VOUCHER

AKV10. Have you heard of the NSW Active Kids Voucher Scheme?

1. Yes

2. No

3. Don’t know [DO NOT READ OUT]

4. Refused [DO NOT READ OUT]

***Interviewer note: Read - The NSW Active Kids Voucher was launched at the beginning of 2018, and provides parents and carers with a $100 rebate to cover the costs of organised sports participation for children aged 5-18 years.***

AKV11. How many children do you have in your family between the ages of 5 and 18 years?

Number field

If don't know, enter 888; If refused, enter 999

AKV12. Did you register any children in the family for a NSW Active Kids Voucher?

1. Yes (GO TO 12.1)

2. No

3. Don't know [DO NOT READ OUT]

4. Refused [DO NOT READ OUT]

AKV12.1 If yes, for how many children?

1. 1

2. 2

3. 3

4. 4

5. 5+

6. Don't know [DO NOT READ OUT]

7. Refused [DO NOT READ OUT]

ALL GO TO AKV13

AKV13. Did you register [randomly selected child] for an Active Kids voucher?

1. Yes

2. No GO TO 32

3. Don't know [DO NOT READ OUT] GO TO 32

4. Refused [DO NOT READ OUT] GO TO 32

AKV14. For [randomly selected child], tell us your main reason for registering with the Active Kids Voucher program?

1. Money/ financial support for activity

2. Support an activity the child usually participates in

3. Try a new activity, instead of usual choice

4. Try a new activity, in addition to usual choice

5. Join an activity the child's friends participate in

6. To encourage the child to do more physical activity

7. Improve child’s health

8. Improve child's fitness

9. Manage child's weight (overweight/obesity)

10. Improve performance in activity

11. Other (Please specify)

12. Don't know [DO NOT READ OUT]

13. Refused [DO NOT READ OUT]

ALL GO TO AKV15

AKV15. Have you redeemed [randomly selected child]’s Active Kids Voucher?

***(Interviewer note: read the following)***

*By redeemed we mean used the voucher to enroll [randomly selected child] into a sport or activity.*

1. Yes GO TO AKV16

2. No GO TO AKV17

3. Don't know [DO NOT READ OUT] GO TO AKV17

4. Refused [DO NOT READ OUT] GO TO AKV17

AKV16. When did you redeem [randomly selected child] voucher?

Open field GO TO AKV18

1. MM/YYYY

Enter 888 for don't know; 999 for refused

AKV17. When do you plan to redeem [randomly selected child] voucher?

Open field GO TO 30

1. MM/YYYY

Enter 888 for don't know; 999 for refused

AKV18. What sport or activity did you redeem [randomly selected child]’s voucher for?

1. Aerobics

2. Athletics, track and field

3. Australian Rules Football

4. Oz tag

5. Basketball

6. Baseball/softball

7. Cheerleading

8. Cricket

9. Cross country / running

10. Crossfit

11. Cycling/mountain biking/bike riding

12. Dancing/Ballet

13. Diving

14. Futsal

15. Golf

16. Gymnastics

17. Horse riding/Equestrian/Polo

18. Hockey

19. Indoor sports (cricket, netball, soccer)

20. League tag

21. Martial arts

22. Netball

23. Ninja Parc

24. Parcor

25. Physie (Physical Culture)

26. Rock climbing

27. Rugby League

28. Rugby Union

29. Skateboarding

30. Soccer

31. Surf life saving

32. Surfing

33. Swimming

34. Tennis

35. Touch Football

36. Water polo

37. Yoga

38. Other (please specify)

39. Don't know [DO NOT READ OUT]

40. Refused [DO NOT READ OUT]

GO TO AKV19

AKV19. In the last week, how many sessions did [randomly selected child] attend for this sport/activity?

***(Interviewer note: read the following)*** *Remember this is ONLY the activity where you used your voucher.*

1. 0 (GO TO AKV21)

2. 1

3. 2

4. 3

5. 4

6. 5

7. 6

8. 7

9. 8+

10. Don't know [DO NOT READ OUT] GO TO AKV21

11. Refused [DO NOT READ OUT] GO TO AKV21

AKV20. On which days in the last week, did [randomly selected child] attend this activity? Tick all that apply

***(Interviewer note: read the following)*** *Remember this is ONLY for the activity which you used Active Kids Voucher.*

1. Monday

2. Tuesday

3. Wednesday

4. Thursday

5. Friday

6. Saturday

7. Sunday

8. Don't know [DO NOT READ OUT]

9. Refused [DO NOT READ OUT]

AKV21. In a typical session, how many minutes/hours did [randomly selected child] spend participating in this sport/activity?

***(Interviewer note: read the following)*** *Remember this is ONLY just the activity where you used your voucher.*

__________________mins / hours

Enter 888 if don't know; 999 if refused

AKV22. In your opinion how has the Active Kids Voucher influenced [randomly selected child] total time being physically active?

1. Increased the child’s activity a lot

2. Increased the child’s activity slightly

3. The child’s activity stayed about the same

4. Decreased the child’s activity slightly

5. Decreased the child’s activity a lot

6. Don't know [DO NOT READ OUT]

7. Refused [DO NOT READ OUT]

AKV23. In the last week, how many sessions in total, of organized sports or physical activities did [randomly selected child] participate in?

***(Interviewer note: read the following)*** *This should include all sessions of structured activity or sport delivered through a club or organization including the activity where you used your active kids voucher and all other sessions as well.*

1. 0 GO TO AKV25

2. 1

3. 2

4. 3

5. 4

6. 5

7. 6

8. 7

9. 8+

10. Don't know [DO NOT READ OUT] GO TO AKV25

11. Refused [DO NOT READ OUT] GO TO AKV25

AKV24. In a typical session, how many minutes/hours did the child spend in the activity?

__________________mins / hours

Enter 888 if don't know; 999 if refused

***Interviewer read: These next few questions require you to rate your level of agreement regarding the NSW Active Kids Voucher Scheme. Please pick the most appropriate answer.***

AKV25. I would not have enrolled my child in organized sport without the support of the Active voucher scheme.

1. Strongly agree

2. Agree

3. Disagree

4. Strongly agree

5. Don't know [DO NOT READ OUT]

6. Refused [DO NOT READ OUT]

AKV26. I support the continuation of the Active Kids Voucher scheme.

1. Strongly agree

2. Agree

3. Disagree

4. Strongly agree

5. Don't know [DO NOT READ OUT]

6. Refused [DO NOT READ OUT]

AKV27. Finding an activity or sporting group that accept the Active Kids Voucher was difficult.

1. Strongly agree

2. Agree

3. Disagree

4. Strongly agree

5. Don't know [DO NOT READ OUT]

6. Refused [DO NOT READ OUT]

AKV28. The Active Kids Voucher supported my child to try a new organized sport.

1. Strongly agree

2. Agree

3. Disagree

4. Strongly agree

5. Don't know [DO NOT READ OUT]

6. Refused [DO NOT READ OUT]

AKV29. It was easy to redeem the Active Kids Voucher.

1. Strongly agree

2. Agree

3. Disagree

4. Strongly agree

5. Don't know [DO NOT READ OUT]

6. Refused [DO NOT READ OUT]

AKV30. It is easy for [randomly selected child] to find and participate in a sport or physical activity?

1. Strongly agree

2. Agree

3. Disagree

4. Strongly agree

5. Don't know [DO NOT READ OUT]

6. Refused [DO NOT READ OUT]

AKV31. In the last 12 months, how much did you pay in total for [randomly selected child] structured physical activity and sport?

***(Interviewer note: read the following)*** *This includes all activities including the activity where you used your voucher and all other activities’.*

1. <$50

2. $50-$149

3. $150-$299

4. $300+

5. Don't know [DO NOT READ OUT]

6. Refused [DO NOT READ OUT]

***These next few questions require you to rate your level of agreement regarding [randomly selected child]’s experience in playing organized sport. Please pick the most appropriate answer.***

AKV32. My child enjoys playing organized sport.

1. Strongly agree

2. Agree

3. Disagree

4. Strongly agree

5. Don't know [DO NOT READ OUT]

6. Refused [DO NOT READ OUT]

AKV33. My child has made new friends through participating in organized sport.

1. Strongly agree

2. Agree

3. Disagree

4. Strongly agree

5. Don't know [DO NOT READ OUT]

6. Refused [DO NOT READ OUT

AKV34. My child is likely to play organized sport next year.

1. Strongly agree

2. Agree

3. Disagree

4. Strongly agree

5. Don't know [DO NOT READ OUT]

6. Refused [DO NOT READ OUT
